# Human umbilical cord mesenchymal stem cells promote carcinoma growth and lymph node metastasis when co-injected with esophageal carcinoma cells in nude mice

**DOI:** 10.1186/s12935-014-0093-9

**Published:** 2014-09-24

**Authors:** Xiaoya Yang, Zhu Li, Yintu Ma, Jun Gao, Surui Liu, Yuhua Gao, Gengyin Wang

**Affiliations:** Blood Transfusion Department, The Bethune International Peace Hospital, Shijiazhuang, 050082 Hebei P R. China

**Keywords:** Umbilical cord, Mesenchymal stem cells, Esophageal carcinoma, Metastasis, Tumor growth

## Abstract

**Background:**

Human umbilical cord blood derived-mesenchymal stem cells (hUCMSCs) offer an attractive alternative to bone marrow-derived MSCs (BMMSCs) for cell-based therapy as it is a less invasive source of biological material. However, limited studies have been conducted with hUCMSCs as compared to BMMSCs. The present study was conducted to evaluate the effects of hUCMSCs in esophageal carcinoma (EC).

**Methods:**

hUCMSCs together with EC cells were transplanted subcutaneously into BALB/c nude mice to observe the effects of hUCMSCs on tumor establishment. hUCMSCs injected through the caudal vein to the mice with pre-established EC to observe the effects of hUCMSCs on tumor outgrowth. In order to elucidate the underlying mechanisms, we also performed in vitro experiments including directly co-culture, transwell assay, proliferation assay and western blotting analysis.

**Results:**

hUCMSCs promoted EC formation in nude mice. In the in vivo model of pre-established EC, intravenously injected hUCMSCs potently promoted tumor growth. When in vitro co-cultured with hUCMSCs, EC cells proliferation increased. After co-cultured with hUCMSCs through transwell system, EC cells showed increased proliferation. Through transwell assay, we also observed that EC cells recruited MSCs, and MSCs promoted EC cells migration and invasion. Western blotting data showed that the expressions of proliferation related proteins Bcl-2, survivin and metastasis related proteins MMP-2 and MMP-9 were up-regulated in the EC cells transwell co-cultured with hUCMSCs.

**Conclusions:**

Our results indicated that hUCMSCs could favor tumor growth in vivo and in vitro. Thus, the exploitation of hUCMSCs in new therapeutic strategies should be cautious under the malignant conditions.

## Background

Mesenchymal stem cells (MSCs) were first identified by Friedenstein and were described as an adherent, fibroblast-like population in the in vitro culture of bone marrow, which were also found to be able to differentiate into bone in vivo [1] Subsequently, the concept expanded, it proved that MSCs are not only bone marrow resident cells but are also found in many other tissues of the body including adipose, umbilical cord, fetal liver, muscle and lung [2-4]. MSCs possess an innate ability for self-renewal and can differentiate into multiple cell types, such as osteocytes, adipocytes, chondrocytes, myocytes, cardiomyocytes, fibroblasts, myofibroblasts, epithelial cells, and neurons [5]. Accumulating studies of the past few years support their use for treating both genetic and acquired human diseases associated to loss of specialized tissues [6,7]. In addition, MSCs have received intensive attention in the field of tumors. Tumor tissue contains abundant growth factors, cytokines and matrix-remodeling proteins, explaining why tumors are likened to wounds that never heal [8]. It has been reported that MSCs migrate to a variety of tumors, this migratory ability points to MSCs as attractive candidates for delivery vehicles of antitumor agents [9,10]. However, several co-injection experiments in animal studies revealed that MSCs promote tumor growth and metastasis [11,12] which would present a serious obstacle to using MSCs as delivery vehicles for anti-cancer therapy. But prior studies on the biology and therapeutic application of human MSCs in human malignancies have reported mixed results. MSCs injected intravenously in a mouse model of Kaposi’s sarcoma were shown to home to sites of tumorigenesis and potently inhibit tumor growth [13]. MSCs have also been shown to have anti-angiogenic effect both in vitro and in mouse models of melanoma [14]. The inconsistent results are clear indicators that the effect of MSCs on tumor cells is poorly understood and need further investigation.

Mesenchymal stem cells used in the experiment are mostly acquired from adult BM. Wharton’s jelly (WJ) of the umbilical cord exhibits the characteristics of stromal cells and is a novel source of mesenchymal stem cells [15]. Mesenchymal stem cells that are derived from WJ of human umbilical cord (hUCMSCs) have been shown to evidence characteristics similar to those of bone marrow stromal cells (BMSCs). Compared to BMMSCs, UCMSCs have many advantages to use in cell-based therapy because of their relatively large ex vivo expansion capacity, low risk of viral infection, lack of donor morbidity, and less pronounced immunogenicity [16-18]. So, it offers an attractive alternative to BMSCs for cell-based therapy. However, the MSCs used in the foundation researches and clinical experiments are mostly acquired from adult BM. Though similarly, there were evidence showed that hUCMSCs have unique properties compared to BMMSCs [19]. However, there is little data on the relationship between hUCMSCs and tumors.

To explore the role of hUCMSCs on tumors, we studied the effects of human hUCMSCs on the esophageal carcinoma (EC) because it occurs with high prevalence in many areas of the world especially in China [20,21]. We investigated the influence of hUCMSCs on EC growth in vivo. We also investigate in vitro co-culture of two different types of EC cell lines with hUCMSCs to explore the mechanism that how hUCMSCs affected tumor growth.

## Results

### Characteristics of hUCMSCs derived from human umbilical cord

**Figure 1:**
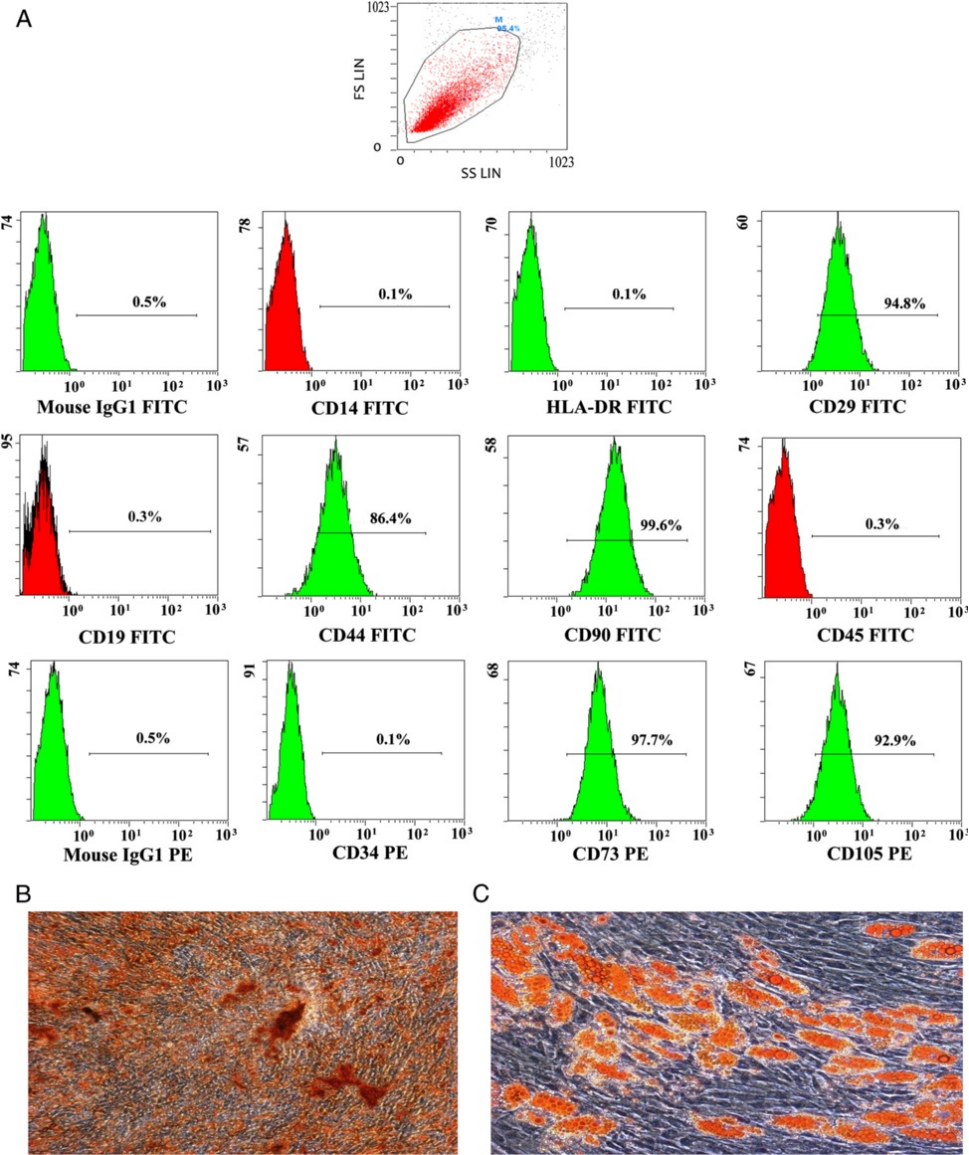
**Characteristics of hUCMSCs derived from human umbilical cord. (A)** Flow cytometric histograms showing the immunophenotype of umbilical vein mesenchymal stem cells. The cells were analyzed by their physical parameters: granularity and size. The gated cells were negative for the hematopoietic line markers CD45, CD14, CD19 and for HLA-DR and CD34. Analyzed cells were positive for CD29, CD73, CD90, CD44 and CD105, which are considered to be markers of mesenchymal stem cells. Isotype controls show non-specific fluorescence. **(B)** HUCMSCs differentiate into osteoblasts. Calcium deposition, indicative of osteoblasts, was stained with Alizarin Red stain. **(C)** HUCMSCs differentiate into adipocytes. The presence of triglycerides, characteristic of adipocytes, was revealed by staining with oil red O.

The isolated clonogenic hUCMSCs were analyzed by the flow cytometry analysis and gated for granularity, size and surface markers. The gated cells were analyzed for the expression of cell membrane proteins markers and found to be negative for the expression of hematopoietic markers such as CD45, CD14, CD19 and also HLA-DR (MHC II) and CD34 (endothelial/hematopoietic stem cell markers), but were positive for CD29, CD44, CD73, CD90 and CD105, which are generally considered for markers of mesenchymal stem cells (Figure 1A). These observations demonstrate that cells that are isolated from WJ of the human umbilical cord have the same surface markers as BMMSCs. The cultured cells were also tested for their potential to differentiate into osteogenic or adipogenic cells prior to use. The effectiveness of osteogenic differentiation was confirmed by histochemical staining for the identification of Ca2+ crystals by Alizarin Red staining (Figure 1B). The effectiveness of adipogenic differentiation was confirmed by histochemical staining for the identification of neutral lipid vacuoles by Oil red O staining (Figure 1C). Thus, the isolated cells met the essential criteria used to define MSCs.

### hUCMSCs favor esophageal cancer growth in vivo

**Figure 2:**
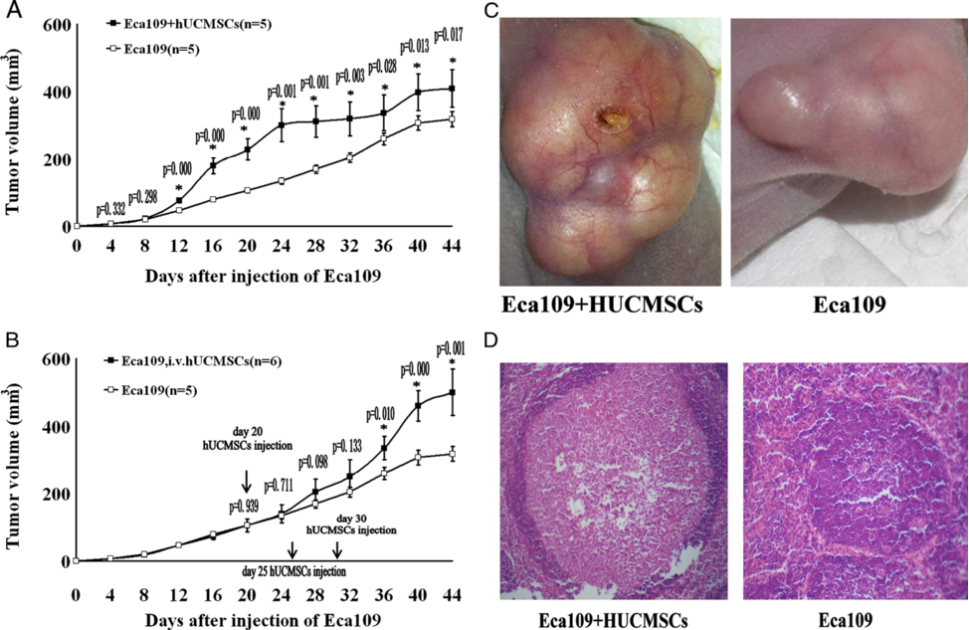
**hUCMSCs favor esophageal cancer growth in vivo. (A)** HUCMSCs promoted the esophageal tumors formation in nude mice. The results were expressed as mean ± S.D. *P < 0.05, Eca109 + hUCMSCs versus Eca109. **(B)** HUCMSCs promoted the pre-established esophageal tumors growth in nude mice. *P < 0.05, Eca109, i.v. hUCMSCs versus Eca109. **(C)** On the 44th day after implantation, the mice were killed. Tumors formed by Eca109 cells admixed with hUCMSCs increased in blood vessel formation in gross analysis. **(D)** Lymph node metastasis was seen in the group that mixed Eca109 cells and hUCMSCs were injected.

To assess the effect of hUCMSCs on esophageal cancer formation *in vivo*, human esophageal squamous cancer cells (Eca109 cell line, 5 × 10^6^ per mouse) were injected subcutaneously into the Armpit region of nude mice alone or mixed with hUCMSCs (5 × 10^6^ per mouse). All of the mice developed growing tumors at the injection sites, and the mice that had received injections of Eca109 and hUCMSCs mixed cells (marked as Eca109 + hUCMSCs group, n = 5) developed bigger tumors than that were observed in the mice injected solely with cancer cells (Eca109 group, n = 5) (Figure 2A). This observation implied that hUCMSCs could promote esophageal cancer formation in nude mice. Because tumor establishment and subsequent tumor growth are distinct biological processes, we sought to determine whether hUCMSCs exerted similar effects in animals with pre-established esophageal tumors. To test this, 5 × 10^6^ Eca109 cells were inoculated in the right Armpit on day 0. Palpable tumors were appreciated in all animals by day 20. A subset of animals was then injected with 5 × 10^6^ hUCMSCs on days 20, 25, and 30 through their caudal vein. These mice were marked as Eca109, iv hUCMSCs group (n = 6). After injection of exogenous hUCMSCs through the caudal vein, the volume of tumor was measured at four-day intervals until sacrifice of the animal. Enhanced tumor volume was observed from day16 after injection of hUCMSCs. (p < 0.05) (Figure 2B). We also found that tumors formed by Eca109 cells admixed with hUCMSCs also increased in blood vessel formation in gross analysis, compared with tumors formed by Eca109 alone (Figure 2C).

Taken together, these observations implied that the formation and development of esophageal cancer were promoted in response to exogenous hUCMSCs.

### Increase in the number of lymph node metastases by injection of Eca109 cells mixed with hUCMSCs

The 16 mice are all alive on day 44. Surviving nude mice were killed. The ipsilateral and contralateral axillary lymph nodes of the nude mice were collected. Every mouse has two ipsilateral axillary lymph nodes and two contralateral axillary lymph nodes. After H&E staining, we found 2 lymph nodes metastases in the 20 lymph nodes of 5 mice that received mixed-cell (Figure 2D). In contrast, lymphatic metastasis was not detected in the other two groups of mice. This result indicated, in a manner, that hUCMSCs enhanced lymph node metastasis of esophageal carcinoma.

### hUCMSCs promote the proliferation of tumor cells in vitro

**Figure 3:**
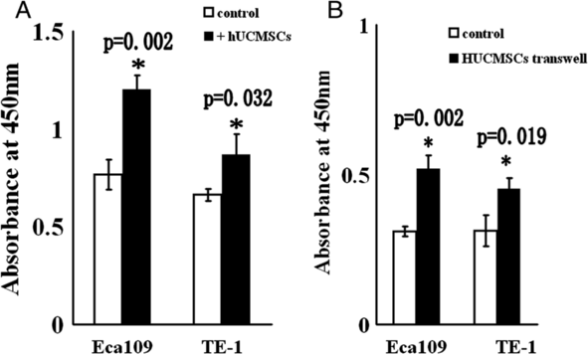
**Proliferation of esophageal cancer cells is promoted by hUCMSCs in vitro. (A)** Esophageal cancer cells and hUCMSCs were performed directly contacting co-culture. Before co-cultured with esophageal cancer cells, hUCMSCs were treated with mitomycin C (20 μg/ml) to prevent cell proliferation. Then esophageal cancer cells were co-cultured with hUCMSCs for 48 hours. Separately cultured cancer cells and mitomycin-treated hUCMSCs were used as control. **(B)** Eca109 and TE-1 cells were co-cultured with hUCMSCs by using the transwell co-culture systems for 48 h, and then, cells were harvested for proliferation assays. The results are shown as mean ± SD of three independent experiments in comparison with that of controls not co-cultured with hUCMSCs (*P < 0.05 versus control).

To understand the mechanisms underlying the in vivo promotion of tumor growth by the injected hUCMSCs, we assessed the proliferation of esophageal squamous cancer cells during their co-culture with hUCMSCs. Considering that using only one esophageal squamous cancer cell line had its limitations in supporting any conclusion, we used two esophageal squamous cancer cell lines, Eca109 and TE-1, in the following experiments. When esophageal cancer cells were directly co-cultured with hUCMSCs in a 24-well plate, the cells proliferation increased significantly when compared with the control group (Figure 3A). To investigate whether hUCMSCs promote tumor cells proliferation through secreting soluble factors, a transwell assay that physically separated hUCMSCs from tumor cells in co-culture system was performed in parallel. Transwell system allows for the effect of soluble factors, but prevents direct cell-cell contact. The statistically significant difference was observed between transwell co-culture group and the control group (Figure 3B). The result suggested that hUCMSCs could promote esophageal cancer cell proliferation through the soluble factors they secrete.

### Esophageal cancer cells attracted hUCMSCs in vitro

**Figure 4:**
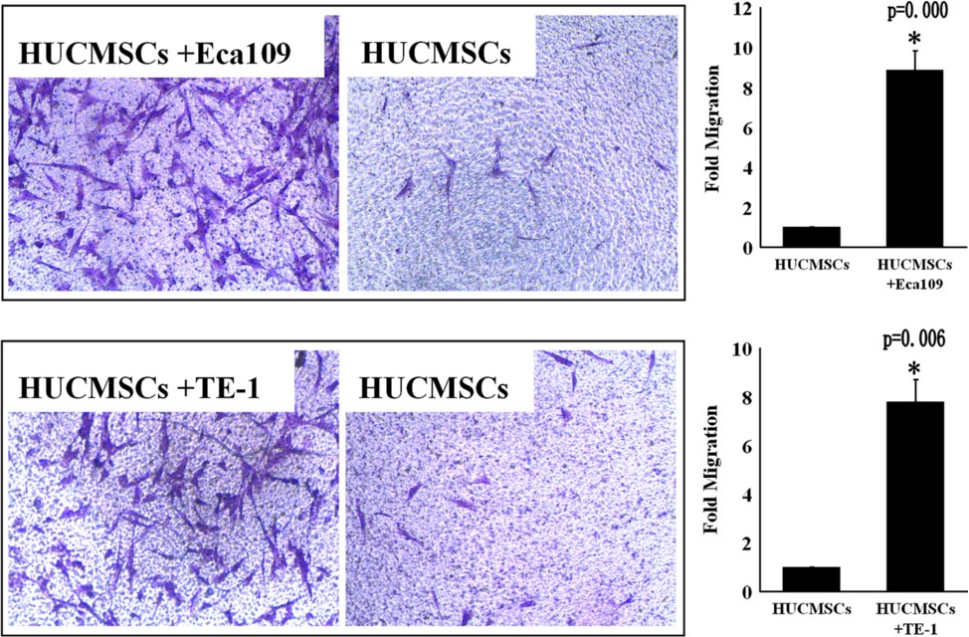
**Esophageal cancer cells attract hUCMSCs in vitro.** The migratory ability of hUCMSCs was analyzed by transwell assay. A significantly greater number of hUCMSCs migrated toward the culture of esophageal cancer cells (Eca109 or TE-1) than toward the medium without esophageal cancer cells. The number of cells was counted in five fields per Transwell membrane. Each group consisted of three duplicates. (∗p < 0.05 versus control).

have the ability to attract hUCMSCs in vitro, migration assay was performed. We found that more hUCMSCs migrated toward the Eca109 or TE-1 cell culture than toward the medium without esophageal cancer cells (Figure 4). Co-cultured with Eca109 cells resulted in an 8.9 fold increased number of migrated cells compared with the controls. Co-cultured with TE-1 cells resulted in a 7.8 fold increased number of migrated hUCMSCs compared with the controls.

### hUCMSCs promoted esophageal cancer cells migration and invasion in vitro

**Figure 5:**
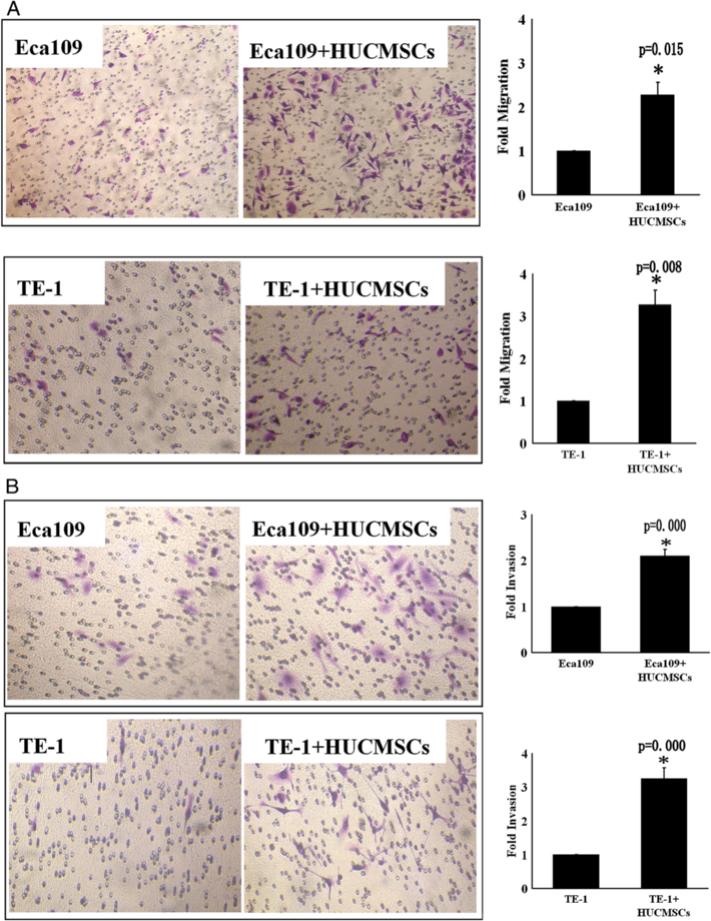
**hUCMSCs promotes migration and invasion of esophageal cancer cells in vitro. (A)** Effects of hUCMSCs on esophageal cancer cells migration. **(B)** Effects of hUCMSCs on esophageal cancer cells invasion. The number of cells was counted in five fields per Transwell membrane. Each group consisted of three duplicates. (∗p < 0.05 versus control).

Migration and invasion of cancer cells is an essential step of cancer metastasis. Since we found that hUCMSCs enhanced the lymph node metastasis of esophageal cancer, the impact of hUCMSCs on esophageal cancer cell migration and invasion was further investigated using transwell assay. Esophageal cancer cells (Eca109 and TE-1 cells) co-cultured with hUCMSCs showed a relative higher ability of migration. Co-cultured with hUCMSCs resulted in an increased number of migrated cells (2.3 fold for Eca109 and 3.3 fold for TE-1) compared with the controls (Figure 5A). In the invasion assay, esophageal cancer cells were seeded into transwell millicell precoated with Matrigel which imitates the extracellular matrix. Cells migrating through the matrix were stained. For Eca109 cells, the ratio of cells invading through matrigel was 110% more in hUCMSCs co-cultured cells than control cells. And co-culturing with hUCMSCs increased TE-1 cells invasion by 220% (Figure 5B).

### Molecular expression changes in esophageal cancer cells co-cultured with hUCMSCs by transwell system

**Figure 6:**
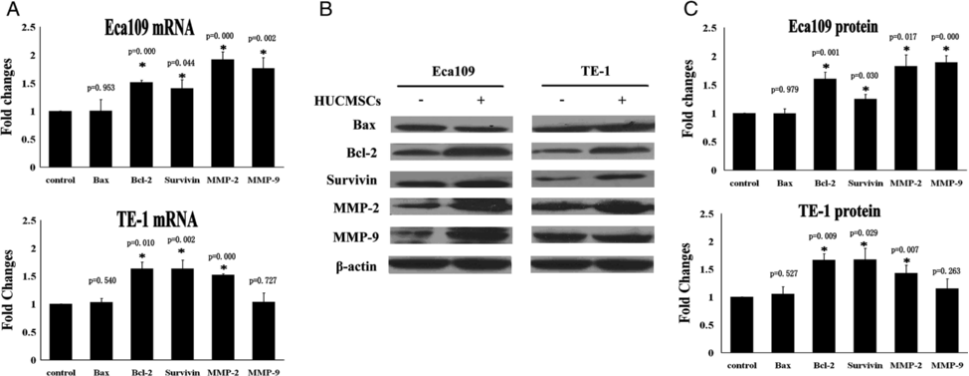
**Molecular changes in esophageal cancer cells co-cultured with hUCMSCs.** The expressions of Bcl-2, Bax, survivin, MMP-2, MMP-9 in Eca109 cells or TE-1 cells were detected by qRT-PCR and Western blotting. Quantitative analysis showed that co-cultured Eca109 cells with hUCMSCs by transwell chamber significantly increased mRNA **(A)** levels and protein **(C)** levels of Bcl-2, survivin, MMP-2 and MMP-9. TE-1 cells co-cultured with hUCMSCs resulted in increased Bcl-2, survivin and MMP-2 mRNA **(A)** levels and protein **(C)** levels as compared to controls. **(B)** Representative Western blotting images. The results were the means ± SD of three independent experiments (*P < 0.05 versus control).

Bcl-2, Bax and survivin are molecules associated with the proliferation and apoptosis of tumor cells. MMP-2 and MMP-9 are the important proteins associated with tumor cells invasion characteristic. In order to find out the molecular mechanism underlying the promotion effect of hUCMSCs on tumor cells in vitro, we investigated the expressions of these proteins in tumor cells by Real-time RT-PCR (Figure 6A) and Western blotting (Figure 6B and C). The results showed that co-cultured Eca109 cells with hUCMSCs by transwell chamber for 48 h resulted in the significant up-regulation of expression levels of Bcl-2, survivin, MMP-2 and MMP-9 as compared to controls. TE-1 cells co-cultured with hUCMSCs for 48 h resulted in increased Bcl-2, survivin and MMP-2 protein levels as compared to controls.

## Discussion

The aim of the current study was to study the interaction between hUCMSCs and esophageal carcinoma. hUCMSCs were transplanted into BALB/c nude mice in an effort to observe the outgrowth of the tumor. The results indicated that all of the groups into which mixed cells were injected evidenced larger tumor size than the groups injected solely with Eca109 cells, thereby indicating that hUCMSCs could favor esophageal tumor formation. Furthermore, we also found that in the mice with pre-established esophageal carcinoma, i.v. injection of hUCMSCs also promoted the tumor growth.

Multiple mechanisms may be responsible for the hUCMSCs induced increase of tumorigenesis and tumor growths. hUCMSCs have been shown to have immunmodulatory action in vivo and in vitro [22,23]. In this experiment, we used nude mice for xenotransplantation. Therefore, increased allogenic tolerance via co-injection with hUCMSCs cannot provide a reasonable explanation for this phenomenon.

In this study, we offer two possible explanations for the enhanced tumor growth in response to hUCMSCs. Firstly, hUCMSCs directly stimulate the growth of esophageal cancer cells. The findings of this study, namely that the mixed culture or cultured by transwell system with hUCMSCs increased the proliferation of esophageal cancer cells in vitro, indicated that hUCMSCs induce the increased proliferation of transplanted tumor cells. It also appears that the promoted effects of hUCMSCs on esophageal carcinoma cells may be both cell-contact dependent as well as mediated via diffusible factors secreted by the hUCMSCs. It was difficult to distinguish tumor cells from hUCMSCs directly cocultured with them, so we only detected the molecular changes in the tumor cells induced by the hUCMSCs-transwell co-culture. The in vitro molecular data showed that the increase in the proliferation of tumor cells were associated with the up-regulation of Bcl-2 and survivin expressions.

In vivo, we observed that tumors formed by Eca109 cells admixed with hUCMSCs increased in blood vessel formation in gross analysis, compared with tumors formed by Eca109 alone. Angiogenesis is critical for tumor growth so that the blood vessel in the tumor environment could provide sufficient nutrients and oxygen to the cells, which are essential for the growth and survival of tumor cells [24]. It is known that MSCs could produce various growth factors that stimulate angiogenesis, [25] so it is possible that enhanced angiogenesis may account for the esophageal tumor growth-promoting effects by hUCMSCs.

In addition, we noticed that in the 20 axillary lymph nodes of 5 mice received mixed-cell 2 lymph nodes metastases were observed, but lymphatic metastasis was not detected in the other two groups of mice. Our results from in vitro assays showed that hUCMSCs promoted migration and invasion ability of esophageal carcinoma cells in vitro. In our in vitro molecular experiment, MMP-2 and MMP-9 were found to be up regulated in EC cells co cultured with hUCMSCs. Since MMP-2 and MMP-9 promotes cell migration and invasion, [26,27] this may be possible mechanism in which hUCMSCs promote esophageal carcinoma cells invasion and thus may be a possible explanation for hUCMSCs promoted the lymph node metastasis of esophageal cancer.

In a paper published in 2010, Li’s results revealed that hMSCs inhibited the proliferation and invasion of Eca-109 cells in vitro [28]. It’ seems that their result is quite different from ours. But the MSCs they investigated were derived from bone marrow. The different sources of MSCs for assessment may be one of the factors accounting for the variability results of pro-tumorigenic or anti-tumorigenic effects. In agreement with this, results of Akimoto’s study demonstrated that umbilical cord blood-derived mesenchymal stem cells inhibit, but adipose tissue-derived mesenchymal stem cells promote glioblastoma multiforme proliferation [29]. So, differences must be considered when choosing a stem cell source for safety in clinical application.

## Conclusions

hUCMSCs offers an attractive alternative to hBMMSCs for cell-based therapy as it is a less invasive source of biological material. However, our results indicated that hUCMSCs could favor esophageal carcinoma growth in vitro and in nude mice model. There were a potential limitation of our experimental designs, as the immune deficient mice does not reflect the real conditions of human beings, whether our results can be extrapolated to humans requires further investigation. However, the data obtained in this study still ringed alarm in using hUCMSCs as therapeutic application for humans in the future. So we suggested that exploitation of hUCMSCs in new therapeutic strategies should be cautious under the malignant conditions, at least under the esophageal carcinoma condition.

## Methods

### Cell culture

The human esophageal cancer cell lines Eca109 and TE-1 was purchased from the Cell Bank of the Chinese Academy of Sciences (Shanghai, China), where they were characterized by mycoplasma detection, DNA-Fingerprinting, isozyme detection and cell vitality detection. The cell lines were maintained in culture as an adherent monolayer in RPMI-1640 (GIBCO) medium supplemented with 10% FBS. All cells were incubated at 37°C in 5% CO_2_ humidified cell culture incubator.

### hUCMSCs preparation

Human umbilical cord samples were collected from 8 healthy donors. Written informed consent was obtained from the pregnant women before labor. The umbilical cord samples were collected in sterile boxes that contained PBS solution. The collected human umbilical cord tissues were washed three times with Ca2+ and Mg2 + −free PBS. They were mechanically cut by scissors in a midline direction and the vessels of the umbilical artery, vein and outlining membrane were dissociated from the WJ. The jelly was then extensively cut into pieces smaller than 0.5 cm^3^. The explants then were cultured in DMEM/F12 (1:1) containing 10% fetal calf serum (FCS). They were left undisturbed for five to seven days to allow for migration of the cells from the explants. The cellular morphology became homogenously spindle-shaped in cultures after 4–8 passages.

### HUCMSCs identification

The ability of MSCs to differentiate into osteoblasts and adipocytes was confirmed prior to use. Osteogenic differentiation was evaluated by calcium deposition staining using the Alizarin Red staining. The induction of adipogenic differentiation was apparent by intracellular accumulation of lipid-rich vacuoles that stained with Oil Red O. The specific surface molecules of HUCMSCs were characterized by flow cytometric analysis. The cells were stained with the following antibodies: CD14-FITC, CD19-ECD, CD29-FITC, CD34-PE, CD44-FITC, CD45-FITC, CD73-PE, CD90-FITC, CD105-PE, HLA-DR-FITC (BD Pharmingen, USA). Thereafter, the cells were analyzed using a Becton Dickinson flow cytometer (Becton Dickinson, San Jose, CA).

### Animal experiments

36 female BALB/c nude mice (4-week-old) were purchased from the Beijing HFK Bioscience CO., LTD. Animals used in this study were maintained in accordance with the Policy of Animal Care and Use Committee of Bethune International Peace Hospital. Animals were housed in micro-isolator cages under sterile conditions and observed for at least 1 week to ensure proper health before study initiation. Animals were injected with 5 × 10^6^ Eca109 cells alone or mixed with an equal number of hUCMSCs, subcutaneously into the Armpit region. Treatment with hUCMSCs was also conducted in animals with pre-established tumors by i.v. tail vein injection at a dose of 5 × 10^6^ per mouse. In some experiments, mice were given additional doses of hUCMSCs by tail vein injection. Tumor growth and progression were monitored by every four days measurements of tumors with calipers. The volume of tumor was calculated by using the following equation as reported previously [30]: volume = Length × Width^2^/2.

### Histopathology and immunhistochemistry

At the indicated time points, the animals were sacrificed. After which tumors were dissected out. The ipsilateral and contralateral axillary lymph nodes of the nude mice were also collected. Then the tumor tissues and the lymph nodes were fixed in 10% formaldehyde and processed using standard methods. The sections were stained using hematoxylin and eosin (H&E) in order to examine the histopathology.

### Cell migration and invasion experiment

For the invasion assay, 24 well Transwell chambers (8.0 μm pore, Corning, USA) were coated with a 50 μl Matrigel (BD, Franklin Lakes). For migration assays, the ECMgel was not needed. Tumor cells were cultured for 24 h in the mixture of tumor cell culture medium and hUCMSCs-conditioned medium (1:1), and in control groups tumor cells were cultured for 24 h in the tumor cell culture medium supplemented with 50% DMEM/F12 (1:1) medium, and then tumor cells were collected and resuspended in the FBS-free RPMI 1640 medium at a concentration of 5 × 10^5^ cell per ml by cell counting for three times. Then, the cell suspensions were put into the upper compartment of the transwell chambers (200 μl/well), and hUCMSCs (5000/well) was put into the lower compartment. After cultured for 24 h, the cells that did not penetrate the polycarbonate membrane at the bottom of the chamber were wiped off with cotton stickers. The membrane was removed and fixed with methanol and stained with Crystal Violet. Five vision fields were randomly selected under microscope and the number of cells that penetrated the membrane was counted. Each group consisted of three duplicates.

The migratory ability of hUCMSCs was also assayed by means of a 24-well microchamber plate with uncoated inserts (8.0 μm pore). Either 2.0 × 10^4^ Eca109 or TE-1 cells in DMEM with 0.5% FBS or medium alone was plated into the lower chambers. After 4 hours of incubation at 37°C, upper chambers containing 2.0 × 10^4^ hUCMSCs in DMEM were set into the lower chambers. Three wells were used for each experiment. After 16 hours of incubation, inserts were fixed with methanol and stained with Crystal Violet. The number of migrating cells was determined as described above.

### Cells co-culture and measurement of cell proliferation

Esophageal cancer cells (Eca109 or TE-1 cells) were cultured either in directly contacting co-cultured or in indirectly co-cultured (transwell system) with hUCMSCs. Esophageal cancer cells and hUCMSCs directly contacting co-cultured was performed as follow: hUCMSCs were first seeded into 24 well plates (5 × 10^4^/ml, 500ul/well). Following 24 hours of incubation, hUCMSCs were found tight attached to the bottom of the well. Before co-cultured with esophageal cancer cells, hUCMSCs were treated with mitomycin C (20 μg/ml) to prevent cell proliferation. Then esophageal cancer cells (5 × 10^4^/ml, 500ul/well) were added to the 24 well plates and co-cultured with hUCMSCs at 37°C for 48 hours. Separately cultured cancer cells and mitomycin-treated hUCMSCs were used as control. Then cell proliferation was determined by Cell Counting Kit-8 (CCK-8, Dojindo, Japan), which allows sensitive colorimetric assays for the determination of the number of viable cells. Before CCK-8 assay, the co-cultured cells and control group cells were detached from the plates. Because the cancer cells can’t easily separated from direct contact co-cultured hUCMSCs, the separately cultured hUCMSCs and cancer cells were put together as controls.

In transwell system, hUCMSCs were physically separated from esophageal cancer cells by a transwell membrane with 0.4-μm pore size (Corning, USA.). hUCMSCs were seeded on upper chamber of 24 well stranswell plate and Eca109 or TE-1 cells were seeded on the lower chamber of transwell plate. The cells were co-cultured for 48 hours. The proliferation of cancer cells was assessed by CCK-8 kit (Dojindo, Japan).

Every group had five wells. All the proliferation experiments were run in triplicate and the results expressed as mean ± SD.

### Real-time RT-PCR

Quantitative PCR was performed using the SYBR Green realtime PCR method. Esophageal cancer cells and hUCMSCs were co-cultured by transwell system for 48 hours. Then, hUCMSCs were collected. Total RNA was isolated from hUCMSCs using an Rneasy Mini Kit (Qiagen, Valencia, CA, USA) and Trizol Reagent (Invitrogen). Quantitative RT-PCR was performed using an ABI 7000 PCR instrument (Applied Biosystems, Foster City, CA, USA). Each sample was tested in triplicate, and the samples obtained from three independent experiments were used for the analysis of relative gene expression using the 2^−△△Ct^ method. The following primers were used for real-time PCR:

- human β-actin, F 5′-cctcgcctttgccgatcc-3′, R 5′-ggatcttcatgaggtagtcagtc-3′;
- human survivin, F 5′-agccctttcaaggaccac-3′, R 5′-gcactttcttcgcagtttcc-3′;
- human Bcl-2, F 5′-tggccagggtcagagttaaa-3′, R 5′-tggcctctcttgcggagta-3′;
- human Bax, F 5′-ttgcttcagggtttcatcca-3′,R 5′-agacactcgctcagcttcttg-3′;
- human MMP-2, F 5′-tctcctgacattgaccttggc-3′, R 5′-caaggtgctggctgagtagatc-3′;
- human MMP-9, F 5′-ttggcgacaagaagtgg-3′, R 5′-gccattcacgtcgtccttat-3′.

### Western blotting

Esophageal cancer cells and hUCMSCs were co-cultured by transwell system for 48 hours. Then, hUCMSCs were collected. Equal amounts of whole cell lysates were resolved by SDS-PAGE and electrotransferred on a PVDF membrane. Primary antibody [anti-human survivin (1:2000; Cell Signaling, USA); anti-human Bcl-2, anti-human BAX and anti-human β-actin (1:1000; Dingguo, China), anti-human MMP-2, anti-human MMP-9 (1:1000; proteintech, USA)] incubation was carried out overnight at 4°C. The immunoreactive signals were detected with an enhanced chemiluminescence kit (Millipore). Quantitative analyses were performed using a Gel Doc 2000 scanner system and Quantity One image analysis software (Bio-Rad).

### Statistical analysis

All statistical analyses were performed using SPSS 13.0 software package. Statistical significance was assessed by comparing mean values (means ± SD). The two-tailed student’s t-test was used to test the probability of significant differences between samples. The significance level was set at P < 0.05.

## Abbreviations

hUCMSCs: Mesenchymal stem cells that are derived from WJ of human umbilical cord
WJ: Wharton’s jelly
EC: Esophageal carcinoma
BMMSCs: Bone marrow-derived Mesenchymal stem cells
MSCs: Mesenchymal stem cells
